# Editorial: New trends in biomimetic tissue and organ modelling

**DOI:** 10.3389/fmedt.2023.1182828

**Published:** 2023-04-17

**Authors:** Camilla Luni, Anna Urciuolo, Jeremy Micah Crook, Carmine Gentile

**Affiliations:** ^1^ Department of Civil, Chemical, Environmental and Materials Engineering (DICAM), University of Bologna, Bologna, Italy; ^2^ Department of Molecular Medicine, University of Padova, Padova, Italy; ^3^ University College London Great Ormond Street Institute of Child Health, London, United Kingdom; ^4^ Neuromuscular Engineering Lab, Institute of Pediatric Research “Città della Speranza”, Padova, Italy; ^5^ Arto Hardy Family Biomedical Innovation Hub, Chris O'Brien Lifehouse, Camperdown, NSW, Australia; ^6^ School of Medical Sciences, Faculty of Medicine and Health, The University of Sydney, Sydney, NSW, Australia; ^7^ Intelligent Polymer Research Institute, AIIM Facility, Innovation Campus, University of Wollongong, Fairy Meadow, NSW, Australia; ^8^ Faculty of Engineering and IT, University of Technology Sydney, Sydney, NSW, Australia; ^9^ Faculty of Medicine and Health, The University of Sydney, Sydney, NSW, Australia

**Keywords:** tissue engineering, biomimetic tissue, stem cell, tissue modelling, organ modeling, organoid, microenvironment, regenerative medicine

**Editorial on the Research Topic** New trends in biomimetic tissue and organ modelling

New and emerging technologies for 3D cell culture and tissue engineering are reshaping biomimetic tissue and organ modelling. Through this Research Topic, contributors provide new insight to a variety of technologies relating to different tissue and organ targets and which control and analyse cellular and extracellular architecture and function for reliable biomimetic modelling and future potential therapy. It is well recognized that tissue architecture is intimately related to tissue function *in vivo*. It follows that engineering the extracellular microenvironment *via* mechanical, topographical, as well as molecular factors dictate cell-matrix and cell-cell interaction and behaviour. To these ends, different natural and synthetic biomaterials, acellular matrices, and micropatterning are being applied towards better reproduction and/or modulation of cell-cell and cell-matrix contacts.

The important role of stem cells as the preferential source of cells for *in vitro* model development is also highlighted. This is due in part to their availability and potential for personalised medicine. In particular, it is recognised that human pluripotent stem cells (hPSCs) can be used to recapitulate *in vitro* the cellular heterogeneity of native tissues. Nevertheless, additional considerations are required to precisely control the environment that stem cells experience to achieve the desired *in vivo*-like phenotypes, further underscoring the important role of the extracellular microenvironment and cell-extracellular matrix (ECM) interaction for optimal tissue ultrastructure, biomechanical features, and bioinductive capabilities.

Mutepfa et al. provided a review of neural stem cell therapy, including the potential for using engineered functionalized biomaterials to treat spinal cord injury (SCI). Despite recent advances in medicine for SCI patients, there have been no definitive findings toward complete functional neurologic recovery. The cellular and structural complexity of SCI underlies the challenge, together with current limitations to using stem cells due to poor control of cell differentiation fates and survival, and integration of transplant cells in the host. Nonetheless, the combination of stem cells with biomaterials presenting mechanical and electroactive properties typical of the spinal cord represents one of the most promising strategies for treating SCI.

The review article by Hong provides an overview of approaches to enhance stem cell performance for tissue engineering using scaffolds, bioinks, membranes, as well as natural and synthetic biomaterials. They highlight the key properties of biomaterials for building a target tissue from stem cells by better engineering the complex *in vivo* cell microenvironment. More specifically, the authors consider biomaterials for engineering skin, bone, spinal cord, vascularisation, trachea and reproductive tract, as well as introducing nanotechnologies for finer architectural engineering and 3D bioprinting for clinical translation.

Yang et al. describe micropatterning technology to investigate tissue patterning, germ layer specification and cell sorting of hPSCs. They showed that hPSCs self-organize to form a radially regionalized neural and non-central nervous system (CNS) ectoderm able to model *in vitro* human ectodermal patterning. Appearance and spatial distribution of the different ectodermal populations derived from hPSCs can be regulated by modulating BMP and WNT signalling within the micropatterning cell culture platforms. Finally, they used their *in vitro* model to dissect the selective cell-sorting behavior of human meso-endoderm cells once seeded onto a pre-patterned ectoderm. They concluded that endoderm, but not mesoderm, segregates from the neural ectoderm, preferentially occupying regions of the non-CNS ectoderm. These findings provide new insight to studying cell-cell interactions occurring during human embryogenesis.

Carraro et al. reviewed the current role of 3D *in vitro* models within the context of skeletal muscle-related pathologies and how they differ from traditional 2D monolayer cultures. The authors described the different cell types present in skeletal muscle and how their spatial organization is recapitulated within *in vitro* 3D constructs. Moreover, they stress the role of the ECM as an essential constituent to engineer biomimetic muscles. The article provides an in depth analysis of the technological challenges for developing 3D *in vitro* models of skeletal muscles. This includes: i) the availability of a reliable cell source; ii) the role played by hydrogels in promoting cellular self-organization, iii) the progress of 3D bioprinting for designing tissue architecture, and iv) the need for mimicking mechanical and electrical cues. In conclusion, the article emphasises the importance of 3D *in vitro* models in reproducing not only the cellular component of the skeletal muscle but also to recapitulate the ECM context for studying specific myopathies.

Finally, the research article by Palmosi et al. reports on the isolation and characterisation of decellularised small intestinal submucosa (dSIS)-derived ECM from pigs to promote cardiac cell function. The dSIS-ECM was tested with human umbilical vein endothelial cells (HUVECs) for live/dead response, as well as to assess tube formation and their ability to promote endothelial cell networks. Finally, proteomic analysis indicated a role played by dSIS on angiogenesis and cell adhesion molecules (i.e., fibronectin). Future *in vivo* studies will be required to further determine the potential translation of dSIS-ECM from the bench to the bedside.

In summary, this Research Topic comprises both novel research and review articles relating to the most recent advances in high-fidelity *in vitro* human tissue and organ modelling. Notwithstanding progress, there remains a need for new strategies to better engineer the complex tissue microenvironment. To this end, a new range of synthetic and/or semi-synthetic biomaterials may help to better tailor features typical of native tissues and organs at the nanoscale, with the potential to also better control their function and application *in vitro* and *in vivo*, being critical for clinical translation.

